# Myristic Acid Produces Anxiolytic-Like Effects in Wistar Rats in the Elevated Plus Maze

**DOI:** 10.1155/2014/492141

**Published:** 2014-09-18

**Authors:** Carlos M. Contreras, Juan Francisco Rodríguez-Landa, Rosa Isela García-Ríos, Jonathan Cueto-Escobedo, Gabriel Guillen-Ruiz, Blandina Bernal-Morales

**Affiliations:** ^1^Unidad Periférica Xalapa, Instituto de Investigaciones Biomédicas, Universidad Nacional Autónoma de México, 91190 Xalapa, VER, Mexico; ^2^Laboratorio de Neurofarmacología, Instituto de Neuroetología, Universidad Veracruzana, Avenida Dr. Luis Castelazo s/n, Colonia Industrial Las Ánimas, 91190 Xalapa, VER, Mexico

## Abstract

A mixture of eight fatty acids (linoleic, palmitic, stearic, myristic, elaidic, lauric, oleic, and palmitoleic acids) at similar concentrations identified in human amniotic fluid produces anxiolytic-like effects comparable to diazepam in Wistar rats. However, individual effects of each fatty acid remain unexplored. In Wistar rats, we evaluated the separate action of each fatty acid at the corresponding concentrations previously found in human amniotic fluid on anxiety-like behaviour. Individual effects were compared with vehicle, an artificial mixture of the same eight fatty acids, and a reference anxiolytic drug (diazepam, 2 mg/kg). Myristic acid, the fatty acid mixture, and diazepam increased the time spent in the open arms of the elevated plus maze and reduced the anxiety index compared with vehicle, without altering general locomotor activity. The other fatty acids had no effect on anxiety-like behaviour, but oleic acid reduced locomotor activity. Additionally, myristic acid produced anxiolytic-like effects only when the concentration corresponded to the one identified in human amniotic fluid (30 *𝜇*g/mL) but did not alter locomotor activity. We conclude that of the eight fatty acids contained in the fatty acid mixture, only myristic acid produces anxiolytic-like effects when administered individually at a similar concentration detected in human amniotic fluid.

## 1. Introduction

Odors from amniotic fluid produce calming reactions and attraction in human newborns [1] and also newborns from other species [2–4], suggesting specific effects of amniotic fluid on emotion and anxiety [5]. An artificial mixture of six fatty acids (capric acid, lauric acid, myristic acid, palmitic acid, oleic acid, and linoleic acid) identified in pig amniotic fluid, colostrum, and maternal milk (*Sus scrofa*, Large White breed) exerts appeasing actions in piglets [6]. An anecdotal report suggested that the same artificial mixture also exerted anxiolytic-like effects in children [6].

In human amniotic fluid, eight fatty acids from colostrum and maternal milk were identified, and an artificial mixture of such human fatty acids produced anxiolytic-like effects that were comparable to diazepam in male and female adult Wistar rats [7, 8]. This fatty acid mixture, based on the content and concentration in human amniotic fluid, included C12:0 (lauric acid), C14:0 (myristic acid), C16:0 (palmitic acid), C16:1 (palmitoleic acid), C18:0 (stearic acid), C18:1* cis* (oleic acid), C18:1* trans* (elaidic acid), and C18:2 (linoleic acid) and produced appetitive responses in human newborns [9]. However, unknown is which of the eight fatty acids contained in human amniotic fluid is mainly responsible for its anxiolytic-like effect.

The present study compared the effects of each of the eight fatty acids against an artificial fatty acid mixture that contained the same eight fatty acids, with diazepam as a reference anxiolytic drug, in Wistar rats subjected to the elevated plus maze [10]. We identified the most potent fatty acid among those contained in the fatty acid mixture in producing changes in the elevated plus maze and tested three different concentrations of this fatty acid in the same test.

## 2. Materials and Methods

### 2.1. Ethics

All of the rat procedures followed the principles of animal care based on the* Guide for the Care and Use of Laboratory Animals* [11] and* Norma Oficial Mexicana para el Cuidado y Uso de Animales de Laboratorio* (NOM-062-ZOO-1999) [12]. The protocol received authorization from the Biomedical Research Institute Ethical Committee (Universidad Nacional Autónoma de México).

### 2.2. Preparation of Fatty Acid Mixture

The fatty acid mixture was prepared according to previous studies that reported its anxiolytic-like effects [7, 8], based on the concentrations found in human amniotic fluid. The mixture consisted of linoleic acid (4.4 mg), palmitoleic acid (7.1 mg), stearic acid (3.7 mg), myristic acid (3.0 mg), elaidic acid (1.5 mg), lauric acid (0.4 mg), oleic acid (8.0 mg), and palmitic acid (15.3 mg) dissolved in 100 mL of vehicle (96% propylene glycol and 4% ethanol) at a temperature <40°C. Each fatty acid that was contained in the mixture was also prepared in the same quantity and individually dissolved in a volume of 100 mL of vehicle, maintaining the same concentration found in human amniotic fluid. All of the fatty acids were of analytical grade and purchased from Sigma-Aldrich (St. Louis, MO, USA). Propylene glycol and ethanol were obtained from J. T. Baker (Xalostoc, MEX, Mexico).

### 2.3. Animals

The experiment included 149 male Wistar rats obtained from a local strain supplied by Harlan (Mexico City, Mexico). The rats were maintained in local housing facilities at a mean temperature of 25°C with a 12 h/12 h light/dark cycle (lights on at 7:00 a.m.). All of the rats included in the study were 11-12 weeks old, weighed 200–250 g, and were housed five rats per cage in acrylic boxes (44 cm width × 33 cm length × 20 cm height) with* ad libitum* access to food (Teklad lab animal diets, Harlan, Indianapolis, IN, USA) and purified water.

### 2.4. Behavioral Tests

#### 2.4.1. Elevated Plus Maze

The elevated plus maze was constructed of wood and situated in a room illuminated at 40 lux. The apparatus consisted of two opposite open and closed arms set in a plus configuration. The dimensions of the open arms were 50 cm × 10 cm (length × width) and the closed arms were 50 cm × 10 cm × 40 cm (length × width × height). The entire maze was elevated 50 cm from the floor. The rats were individually placed at the center of the maze, facing an open arm, and the time spent on and number of entries into the open arms were recorded [13]. The total number of entries (open arms + closed arms) and percentage of open arm entries ([open entries]/[total entries] × 100) were calculated. Additionally, the anxiety index was calculated according to Cohen et al. [14] as follows: Anxiety Index = 1 − [([Open arm time/Test duration] + [Open arms entries/Total number of entries])/2].

#### 2.4.2. Locomotor Activity Test

To evaluate the effects of the treatments on spontaneous locomotor activity and discard the possibility of hypoactivity or hyperactivity attributable to the treatments that could influence performance in the elevated plus maze, a 5 min locomotor activity test was performed after the elevated plus maze test in another room (40 lux). We used an automated locomotor activity monitor (Acti-Track v2.7.10, PanLab, S.L., Instrument, Barcelona, Spain) constructed of a Perspex box (45 cm × 45 cm) with 35 cm high walls. The apparatus was elevated 3 cm above the box frame floor. A total of 32 infrared beams, 16 on each perpendicular wall, were connected to an interface (LE 8811, LSI Letica Scientific Instrument, Barcelona, Spain) and subsequently to a computer. For data analysis, the floor of the cage was virtually divided into five zones (four peripheral and one central), and we measured the number of entries into each zone (crossings), total resting time, and total activity time during the 5 min test as indicators of locomotion. No other behaviors, such as rearing or grooming, were evaluated. Because of the relatively small cage, we did not compare central versus peripheral exploration.

After each experimental session, the elevated plus maze and locomotor activity box were carefully cleaned and deodorized with a 5% ethanol cleaning solution. Approximately 5 min elapsed between each test to allow the scent of the substances to dissipate.

### 2.5. Experimental Groups and Treatments

The rats were assigned to 11 experimental groups: (i) vehicle (*n* = 10), (ii) fatty acid mixture (FAT-M; *n* = 9), (iii) linoleic acid (44 *μ*g/rat, *n* = 10), (iv) palmitoleic acid (71 *μ*g/rat, *n* = 10), (v) stearic acid (37 *μ*g/rat, *n* = 10), (vi) myristic acid (30 *μ*g/rat, *n* = 10), (vii) elaidic acid (15 *μ*g/rat, *n* = 10), (viii) lauric acid (4 *μ*g/rat, *n* = 10), (ix) oleic acid (80 *μ*g/rat, *n* = 10), (x) palmitic acid (153 *μ*g/rat, *n* = 10), and (xi) diazepam (2 mg/kg, *n* = 10). All of the injections were administered subcutaneously in a volume of 1 mL/rat, with the exception of diazepam, which was administered intraperitoneally. The rats were first tested in the elevated plus maze (5 min) and then in the locomotor activity test (5 min). One hour elapsed between the injections of substances and the beginning of the behavioral tests.

In another group of rats, we evaluated three concentrations of the fatty acid that produced significant anxiety-like behavior in the elevated plus maze. Four experimental groups were included: vehicle (*n* = 10), one-third the concentration of the fatty acid found in human amniotic fluid (*n* = 10), the full concentration of the fatty acid found in amniotic fluid (*n* = 10), and double the concentration of the fatty acid found in human amniotic fluid (*n* = 10). All of the injections were administered subcutaneously in a volume of 1 mL/rat, 1 h before the behavioral tests. The rats were tested first in the elevated plus maze (5 min) and then in the locomotor activity test (5 min).

### 2.6. Statistical Analysis

To analyze the treatment data, one-way analysis of variance (ANOVA) was used. Significant effects in the ANOVA were followed by Dunnett's* post hoc* test. The results are expressed as mean ± standard error. Values of *P* ≤ 0.05 were considered statistically significant.

## 3. Results

### 3.1. Eight Fatty Acids

#### 3.1.1. Elevated Plus Maze

**Figure 1:**
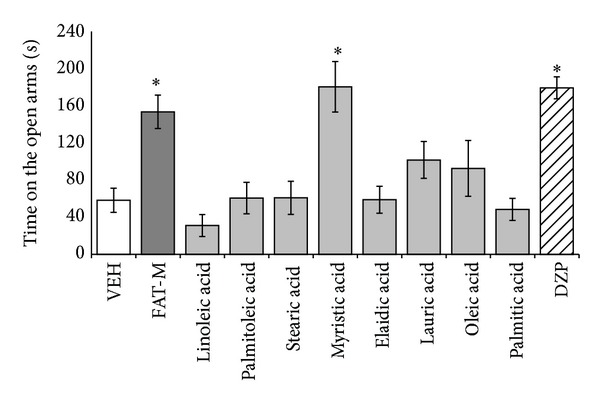
Elevated plus maze. Total time spent on open arms significantly increased in the fatty acid mixture (FAT-M), myristic acid, and diazepam (DZP) groups, compared with vehicle group (VEH). *P < 0.05 versus vehicle group. One-way ANOVA; Dunnett* post hoc* test.

Significant differences were found between treatments (*F*_10,98_ = 8.435; *P* < 0.001) in the time spent on the open arms. The* post hoc* test showed that the time spent on the open arms was longer (*P* < 0.05) in the diazepam, fatty acid mixture, and myristic acid groups compared with the vehicle group. The time spent on the open arms was similar in the vehicle group and individual fatty acid groups, with the exception of the myristic acid group (Figure 1).

Table 1 illustrates the number of open arm entries, percentage of open arm entries, and total number of entries into the open and closed arms. The number of open arm entries was significantly different between treatments (*F*_10,98_ = 6.160; *P* < 0.001). The* post hoc* test showed that the number of open arm entries was higher (*P* < 0.05) in the fatty acid mixture group and diazepam group than in the vehicle group, but none of the individual fatty acids produced changes compared to vehicle. Likewise, the treatments produced significant changes (*F*_10,98_ = 11.579; *P* < 0.001) in the percentage of open arm entries. Only the fatty acid mixture group and diazepam group exhibited a higher percentage of open arm entries than the vehicle group, but none of the individual fatty acids significantly modified this variable. The treatments did not produce any significant changes (*F*_10,98_ = 1.592; *P* = 0.120) in the total number of entries into either the open or closed arms.

**Figure 2:**
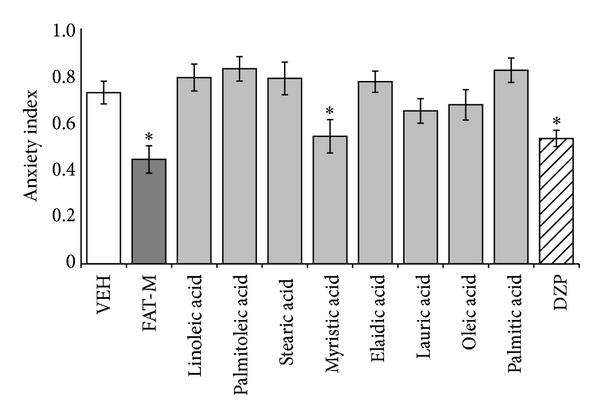
Elevated plus maze. Anxiety index significantly was reduced in the fatty acid mixture (FAT-M), myristic acid, and diazepam (DZP) groups, compared with vehicle group (VEH). *P < 0.05 versus vehicle group. One-way ANOVA; Dunnett* post hoc* test.

The analysis of the anxiety index in the elevated plus maze revealed significant differences between treatments (*F*_10,98_ = 5.160; *P* < 0.001). The* post hoc* test showed that the anxiety index in the diazepam, fatty acid mixture, and myristic acid groups was significantly smaller (*P* < 0.05) than in the vehicle group. No other significant differences were observed (Figure 2).

#### 3.1.2. Locomotor Activity Test

The number of crossings, activity time, and resting time in the locomotor activity test are presented in Table 2. Significant differences were found between treatments in the number of crossings (*F*_10,98_ = 2.115; *P* < 0.03), activity time (*F*_10,98_ = 3.812; *P* < 0.001), and resting time (*F*_10,98_ = 3.807; *P* < 0.001). The* post hoc* test revealed that oleic acid significantly (*P* < 0.05) reduced the number of crossings and activity time compared with all of the other groups.

### 3.2. Myristic Acid

#### 3.2.1. Elevated Plus Maze

**Figure 3:**
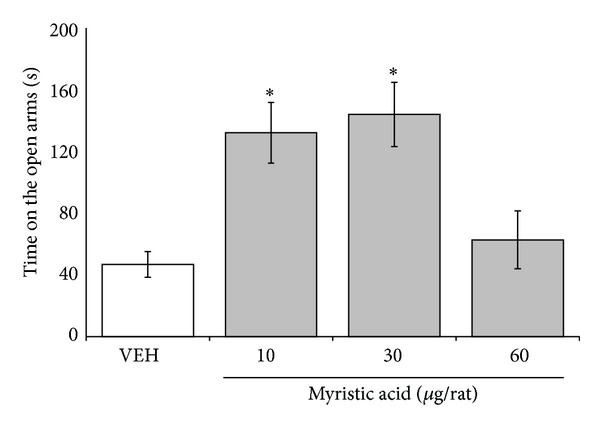
Elevated plus maze. Total time spent on open arms significantly increased in 10 and 30 *µ*g/rat of myristic acid, compared with vehicle group (VEH). *P < 0.05 versus vehicle group. One-way ANOVA; Dunnett* post hoc* test.

Significant differences were found in the time spent on the open arms between the groups treated with different concentrations of myristic acid (*F*_3,36_ = 7.672; *P* < 0.001). The* post hoc* test showed that the time spent on the open arms was longer (*P* < 0.05) in the one-third concentration (10 *μ*g/rat) and full concentration (30 *μ*g/rat) groups compared with the vehicle group (Figure 3), but the double concentration (60 *μ*g/rat) group exhibited a similar action to the vehicle group.

A significant effect of myristic acid on the number of entries into the open arms was observed (*F*_3,36_ = 2.838; *P* < 0.05). The* post hoc* test showed that the number of entries into the open arms was higher (*P* < 0.05) in the one-third concentration group compared with the vehicle group. No significant differences were found in the total number of arm entries (open + closed) and percentage of entries into the open arms (Table 3).

**Figure 4:**
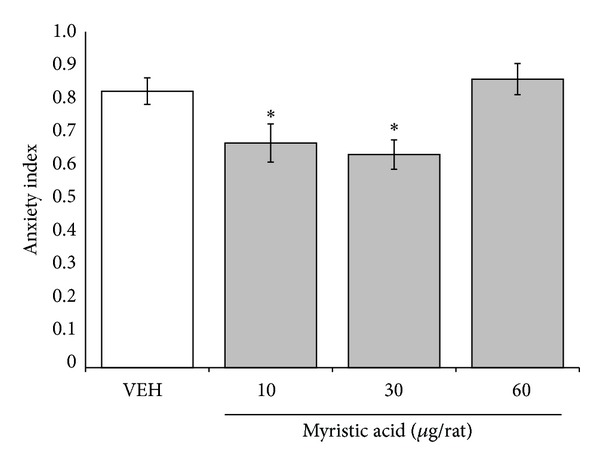
Elevated plus maze. Anxiety index significantly was reduced in 10 and 30 *µ*g/rat of myristic acid groups, compared with vehicle group (VEH). *P < 0.05 versus vehicle group. One-way ANOVA; Dunnett* post hoc* test.

Finally, the analysis of the anxiety index in the elevated plus maze revealed significant differences between the myristic acid concentrations (*F*_3,36_ = 5.607; *P* < 0.003). The* post hoc* test showed that the anxiety index in the one-third and full concentration groups was significantly smaller (*P* < 0.05) than in the vehicle group. No significant differences in the anxiety index were found between the double concentration group and vehicle group (Figure 4).

#### 3.2.2. Locomotor Activity Test

The number of crossings, activity, and resting time in the locomotor activity test are presented in Table 4. Significant differences were detected between the concentrations of myristic acid in the number of crossings (*F*_3,36_ = 7.385; *P* < 0.001), activity time (*F*_3,36_ = 12.629; *P* < 0.001), and resting time (*F*_3,36_ = 11.481; *P* < 0.001). The* post hoc* test revealed that the one-third concentration group exhibited a significant (*P* < 0.05) increase in activity time and decrease in resting time in the locomotor activity test compared with the vehicle group. Additionally, the double concentration group exhibited a significant decrease in activity compared with the vehicle group (Table 4).

## 4. Discussion

The present study compared the anxiolytic-like effects of eight fatty acids separately against diazepam and a mixture of the same eight fatty acids in adult male Wistar rats that were subjected to the elevated plus maze. Myristic acid was the only fatty acid that individually produced comparable effects to both diazepam and the fatty acid mixture, including a longer time spent on the open arms (approximately twofold longer compared with vehicle) and a smaller anxiety index (approximately one-third smaller compared with vehicle). Three concentrations of myristic acid were then evaluated. Anxiolytic actions were observed only with the full concentration of myristic acid found in human amniotic fluid and not with lower or higher concentrations.

The elevated plus maze is a widely used behavioral test that assesses anxiety-like behavior and the anxiogenic-like or anxiolytic-like effects of pharmacological agents [13, 15]. Rodents that display anxiety-like behavior in the elevated plus maze usually exhibit a reduction of the time spent on the open arms [16, 17], whereas animals that are treated with *γ*-aminobutyric acid (GABA), agonist anxiolytic compounds, including benzodiazepines (e.g., diazepam), and some neurosteroids (e.g., progesterone and allopregnanolone) exhibit an increase in the total time spent on the open arms [18, 19]. Other variables that are evaluated in the elevated plus maze (i.e., number of entries into the open arms and total number of entries into the arms) are integrated in an anxiety index, with values that range from 0 to 1. An increase in the anxiety index indicates higher anxiety-like behavior. The anxiety index coalesces data for each of the individual parameters of exploratory behavior in the elevated plus maze and indicates an overall tendency [14]. An anxiolytic-like effect of myristic acid was confirmed by calculating the anxiety index, which was significantly lower compared with vehicle and was similar to the fatty acid mixture and diazepam.

Other models are used to experimentally study the anxiolytic actions of drugs, such as the light/dark test [20, 21] and defensive burying test [22, 23], among others. The current trend is to use two or more models to determine anxiolytic actions, including the open field test using cage dimensions that are appropriate to the experimental subject [24–27]. However, each test possesses a particular correspondence to some types of anxiety. The defensive burying test explores generalized anxiety [23], whereas the open field test may be used to explore neophobia [28]. We selected the elevated plus maze. This test explores the conflict between the natural behavior of rodents to explore new spaces and avoid open/illuminated spaces. Thus, behavior in the elevated plus maze may reflect some types of phobias to open spaces [29, 30]. The evaluation of some anxiolytic drugs requires the use of only one test (i.e., elevated plus maze or defensive burying test), such as the benzodiazepines chlordiazepoxide and diazepam [31–33], selective serotonin reuptake inhibitor fluoxetine [34, 35], and neurosteroids estradiol, progesterone, and allopregnanolone [36–38]. In the present study, myristic acid produced some anxiolytic actions in the elevated plus maze comparable to diazepam, resembling the actions of benzodiazepine anxiolytics, fluoxetine, or some neurosteroids in this behavioral test [33, 35, 38]. The effects of myristic acid in the elevated plus maze did not reproduce all the actions of a typical anxiolytic drug (i.e., diazepam), suggesting that its behavioral profile corresponds to a weak anxiolytic drug.

In the present study, the locomotor activity test was conducted after the elevated plus maze test, allowing us to identify possible changes in motor activity associated with the treatments that may influence performance in the elevated plus maze. In the locomotor activity test, similar to diazepam [19, 39], the fatty acid mixture did not produce any significant modifications of motor activity [7]. Therefore, the effect of myristic acid in the elevated plus maze may be considered an anxiolytic-like effect, which supports at least one clinical study [40], in which a negative correlation was found between anxiety traits and myristic acid levels in adipose tissue (i.e., higher myristic acid levels in adipose tissue were associated with lower trait anxiety).

One-third the concentration of myristic acid in amniotic fluid produced some changes that suggested an increase in locomotor activity, including an increased number of entries in the elevated plus maze. This low concentration also increased activity time and reduced resting time in the open field test. The one-third concentration of myristic acid did not produce an evident anxiolytic-like action in the elevated plus maze but rather produced locomotor hyperactivity. The double concentration of myristic acid clearly reduced activity on the open field test. Therefore, myristic acid seemingly exerts actions that are similar to other central nervous system depressants (e.g., alcohol) that produce motor hyperactivity in the open field test at low doses [41, 42] but motor hypoactivity at higher doses [43–45]. These observations suggest that myristic acid acts as a central nervous system depressant and exerts anxiolytic actions only at the concentration found in human amniotic fluid.

A possible limitation of the present study was that we did not explore the mechanism of action of the anxiolytic-like effect of myristic acid. This fatty acid appears to be an important cellular component because numerous proteins require myristoylation to exert their biological effects on transduction pathways, vesicular trafficking, and structural positioning [46]. Fatty acids have been proposed to be able to produce conformational changes in ion channels and alter ion conductance [47]. However, the main action of some fatty acids (e.g., oleic acid, linoleic acid, ricinoleic acid, and arachidonic acid) appears to occur through the regulation of chloride ion channels [48]. Oleic acid increases the affinity of agonists for the benzodiazepine site of GABA_*A*_ receptors [49], thus modulating the opening of chloride channels. This may be related to the observations in the present study, in which oleic acid, although devoid of anxiolytic-like effects, produced hypoactivity in the locomotor activity test, suggesting a GABAergic effect. Myristic acid may also contribute to modulating the opening of GABA_*A*_ receptor chloride channels. The anxiolytic actions of an artificial mixture that contained this fatty acid were previously shown to be blocked by picrotoxin, suggesting that the mixture of fatty acids as a whole exerted its actions on GABA_*A*_ receptor chloride channels [50]. The possible participation of chlorine channels in the effects of myristic acid and oleic acid requires further exploration.

Myristic acid is a saturated fatty acid (C16:0) that has been identified in nutmeg (*Myristica fragrans*). It is also contained in palm kernel oil and coconut butterfat and in lower amounts in many other animal fats, including bovine and human milk and colostrum [7, 51, 52]. The general profile of myristic acid does not fulfill the profiles of diazepam or the artificial fatty acid mixture. The present results showed that the time spent on the open arms in the elevated plus maze was longer in the myristic acid, fatty acid mixture, and diazepam groups than in the vehicle group. However, the number and percentage of entries into the open arms were increased only by the fatty acid mixture and diazepam and not by myristic acid, indicating the low potency of myristic acid as an anxiolytic agent and strongly suggesting the possibility of interactions among fatty acids to produce a full anxiolytic effect.

In conclusion, myristic acid seemingly exerted anxiolytic-like effects, producing comparable actions to diazepam in some variables of the elevated plus maze without altering spontaneous locomotion.

## Figures and Tables

**Table 1:** Number of open arm entries, percentage of open arm entries, and total number of entries into open and close arms in elevated plus maze.

Treatment	Open arms entries (*n*)	Open arms entries (%)	Total entries to arms (*n*)
Vehicle	4.10 ± 0.69	30.08 ± 3.60	12.80 ± 1.71
FAT-M	7.88 ± 0.92∗	59.53 ± 6.25∗	13.55 ± 1.83
Linoleic acid	2.00 ± 0.61	19.14 ± 4.76	7.50 ± 1.55
Palmitoleic acid	4.30 ± 1.49	24.22 ± 5.66	10.90 ± 2.27
Stearic acid	3.10 ± 0.88	21.83 ± 5.03	11.50 ± 1.66
Myristic acid	5.30 ± 1.01	44.72 ± 2.23	12.00 ± 2.25
Elaidic acid	3.60 ± 0.71	25.52 ± 4.67	12.80 ± 1.20
Lauric acid	5.10 ± 0.97	35.96 ± 4.40	13.10 ± 2.26
Oleic acid	4.10 ± 0.78	36.04 ± 4.36	10.50 ± 0.92
Palmitic acid	2.60 ± 0.60	26.07 ± 4.87	8.30 ± 1.33
Diazepam	9.70 ± 1.00∗	69.07 ± 4.95∗	14.30 ± 0.85

Values are expressed as mean ± standard error from each variable. *P < 0.05 versus vehicle group. FAT-M: fatty acid mixture group. One-way ANOVA; Dunnett *post hoc* test.

**Table 2:** Number of crossing, activity time, and resting time in the locomotor activity test.

Treatment	Crossing (*n*)	Activity time (s)	Resting time (s)
Vehicle	41.10 ± 6.02	99.36 ± 4.95	206.84 ± 6.37
FAT-M	47.33 ± 7.33	108.63 ± 8.29	189.17 ± 8.09
Linoleic acid	29.30 ± 2.79	74.34 ± 10.12	225.65 ± 10.12
Palmitoleic acid	46.00 ± 7.21	97.43 ± 9.54	202.52 ± 9.54
Stearic acid	40.60 ± 5.57	89.33 ± 6.87	210.64 ± 6.87
Myristic acid	45.90 ± 7.05	110.24 ± 13.25	189.22 ± 13.09
Elaidic acid	33.30 ± 4.71	85.71 ± 6.90	214.27 ± 6.89
Lauric acid	46.20 ± 11.54	108.25 ± 11.93	191.75 ± 11.93
Oleic acid	22.50 ± 3.89∗	54.92 ± 5.84∗	245.08 ± 5.84∗
Palmitic acid	26.50 ± 3.47	76.86 ± 6.99	223.10 ± 6.99
Diazepam	31.40 ± 2.58	100.66 ± 8.77	199.33 ± 8.77

Values are expressed as mean ± standard error from each variable. *P < 0.05 versus vehicle group. FAT-M: fatty acid mixture group. One-way ANOVA; Dunnett *post hoc* test.

**Table 3:** Number of open arm entries, percentage of open arm entries, and total number of entries into open and close arms in myristic acid treated rats in elevated plus maze.

Treatment	Open arms entries (*n*)	Open arms entries (%)	Total entries to arms (*n*)
Vehicle	3.90 ± 0.54	31.84 ± 2.97	12.30 ± 1.43
Myristic acid			
10 *μ*g/rat	6.90 ± 0.70∗	40.80 ± 4.08	18.50 ± 0.84
30 *μ*g/rat	5.40 ± 0.76	40.87 ± 4.85	13.90 ± 1.47
60 *μ*g/rat	4.10 ± 0.93	26.70 ± 3.62	14.10 ± 2.66

Values are expressed as mean ± standard error from each variable. *P < 0.05 versus vehicle group. One-way ANOVA; Dunnett *post hoc* test.

**Table 4:** Number of crossing, activity time, and resting time in myristic acid treated rats in locomotor activity test.

Treatment	Crossing (*n*)	Activity time (s)	Resting time (s)
Vehicle	51.30 ± 7.78	108.98 ± 7.78	191.02 ± 7.76
Myristic acid			
10 *μ*g/rat	68.50 ± 8.79	147.38 ± 7.70∗	152.64 ± 7.67∗
30 *μ*g/rat	29.80 ± 5.03∗	93.36 ± 8.44	206.62 ± 8.40
60 *μ*g/rat	29.30 ± 5.45∗	79.68 ± 8.93∗	214.78 ± 8.65

Values are expressed as mean ± standard error from each variable. *P < 0.05 versus vehicle group. FAT-M: fatty acid mixture group. One-way ANOVA; Dunnett *post hoc* test.
